# Food Insecurity and Engagement in Transactional Sex Among Female Secondary Students in Rwanda

**DOI:** 10.1007/s10461-023-04173-2

**Published:** 2023-10-04

**Authors:** Lila A. Sheira, Jonathan Izudi, Emmyson Gatare, Laura Packel, Laetitia Kayitesi, Felix Sayinzoga, Rebecca Hope, Sandra I. McCoy

**Affiliations:** 1 Division of Epidemiology, School of Public Health, University of California, Berkeley, Berkeley, USA; 2 African Population and Health Research Center, Nairobi, Kenya; 3 YLabs, Kigali, Rwanda; 4 Rwanda Biomedical Center, Ministry of Health, Kigali, Rwanda; 5 YLabs, Berkeley, CA USA

**Keywords:** Food security, Adolescent girls, Young women, Transactional sex, Rwanda

## Abstract

The relationship between food insecurity and transactional sex is well recognized, but less is known about this relationship among adolescents. We analyzed cross-sectional baseline data from 3,130 female secondary students aged 12–19 enrolled in a three-arm, cluster randomized controlled trial to examine the association between food insecurity and transactional sex. The explanatory variable was food security and the outcome was ever engaging in transactional sex. Over one quarter (28.7%) reported any food insecurity and 1.9% of all participants (9.6% of sexually active participants) reported ever engaging in transactional sex. In adjusted models, ever experiencing any food insecurity was associated with a higher prevalence of ever transactional sex (PR: 1.60; 95% CI: 1.02, 2.49) compared to little to no food insecurity. These results provide insight into potential predictors of higher-risk sexual behavior in Rwanda; they also provide policy-makers with populations with whom to intervene on upstream determinants of transactional sex, notably poverty and food insecurity.

## Resumen

La inseguridad alimentaria y el sexo transaccional están poco estudiados entre las adolescentes ruandesas. Analizamos datos de referencia transversales de 3,130 estudiantes secundarias de 12 a 19 años de edad inscritas en un ensayo controlado aleatorio por grupos de tres brazos para examinar la asociación entre la inseguridad alimentaria y el sexo transaccional. La variable independiente fue la seguridad alimentaria y la variable dependiente fue participation en sexo transaccional por lo menos una vez. Más de una cuarta parte (28,7%) informó alguna inseguridad alimentaria y el 1,9% de todos los participantes (9,6% de los participantes sexualmente activos) informaron haber tenido relaciones sexuales transaccionales alguna vez. En modelos ajustados, experimentar alguna inseguridad alimentaria alguna vez se asoció con una mayor relación sexual transaccional (PR: 1,60; IC del 95%: 1,02, 2,49) en comparación con poca o ninguna inseguridad alimentaria. Estos resultados brindan información sobre posibles predictores de comportamiento sexual de mayor riesgo en Ruanda; también brindan a los formuladores de políticas poblaciones para intervenir en los determinantes anteriores del sexo transaccional, en particular la pobreza y la inseguridad alimentaria.

## Introduction

Food insecurity (FI), defined as “the uncertain or limited availability of nutritionally adequate or safe food or the inability to procure food in socially acceptable ways,” [1] affected nearly 30% of the global population in 2021. [2] This crisis is heighted on the African continent, where 57.9% of households experienced any FI and nearly 1 out of every 4 households experienced severe FI in 2021. [2] Further, in Sub-Saharan Africa and specifically East Africa, 63% and 67% of households, respectively, experienced FI in 2021. [2] Within households, adolescent girls and young women (AGYW) often bear a disproportionate burden of FI. [3].

Approximately 81% of Rwandans are food secure although the U.N. Food and Agricultural Organization estimates that 35% of Rwandans are undernourished, a distinct but related concept to food security. [4] FI is a well-documented driver of behaviors that place women, particularly AGYW, at a higher risk for sexually transmitted infections, such as HIV, and unplanned pregnancy. [5–7] Despite a low prevalence of HIV in the general Rwandan population, comprehensive knowledge of HIV declined from 65 to 59% among Rwandan AGYW in the most recent Rwandan Demographic and Health Survey, [8] which also reported that half of sexually active AGYW were tested for HIV in the previous year. [8] FI has a demonstrated association with transactional sex, [9] a known driver of HIV. [6] Transactional sex is prevalent regionally, ranging from 14% among a sample of AGYW in South Africa [10] to 25% among university aged females in Uganda; [11] some estimates have neared 80%. [12] Nevertheless, the relationship between FI and transactional sex has been understudied among adolescents and specifically in Rwanda. Therefore and in this study, we examined the association between FI and transactional sex among in-school AGYW in Rwanda.

## Methods

Data used for this study are from the baseline survey conducted among AGYW in secondary schools enrolled in a three-arm, cluster Hybrid Trial Type 2 Effectiveness-Implementation study [13] to evaluate the impact of CyberRwanda, a digital health intervention, which has been described elsewhere. [14].

Eligible secondary schools included: (1) those that were located ≤ 4.5 km of a participating pharmacy (as the intervention links to pharmacies); (2) were located ≥ 1.5 km from another secondary school to reduce spillover; (3) had ≥ 150 students; (4) were a day school; (5) had a minimum of 150 students; and (6) were willing to participate (determined by school leadership).

Students < 18 years of age were invited to participate via parental consent forms sent home. We created a sampling frame among students who had parental consent or those who self-consented (age 18–19). The study team randomly sampled approximately fifty students per sex per school, resulting in 6,079 eligible students at study baseline (February-May 2021). Baseline survey data from all 3,130 AGYW aged 12–19 were analyzed in this study.

Ethical approval was given by the Rwanda National Ethics Committee and the University of California, Berkeley Committee for Protection of Human Subjects. The trial is registered at the US National Clinical trials registry site (Registration # NCT04198272).

### Variables and Measurements

The outcome of interest was (ever) transactional sex, defined as an affirmative response to the question “have you ever had sex in exchange for money, food, gifts, drugs, alcohol, shelter, or other goods?”. AGYW who reported never having engaged in transactional sex, as well as those who had never engaged in any sex were coded as “no”. Participants with other non-affirmative responses (“don’t know” [n = 18], “refuse to answer” [n = 15], and “NA” [n = 1]) were excluded.

The explanatory variable was household FI over the past thirty days, measured via the widely used and validated six-item household hunger scale. [15] The scale was categorized per standard scoring algorithms into: (1) a binary variable (any FI compared to little to no FI [reference]) and (2) a three-category variable (little to no [reference], moderate, and severe hunger) and analyzed in two models. Missing food security status data was minimal (1.5%) so a complete case analysis was conducted. The food security scale had a Cronbach’s alpha of 0.89 in the entire sample, indicating high internal consistency.

Potential confounders included were based on *a priori* knowledge of the relationship between food security and sexual health, including age of the participant and educational attainment of the participant’s mother (primary or less [reference], completed primary or greater, and unknown). Further, a wealth index was developed using household assets and dwelling characteristics (i.e. floor material) per standard scoring. [16].

### Statistical Analysis

We summarized the participant demographic characteristics and assessed bivariate associations between food security status and transactional sex. We implemented covariate-adjusted generalized linear models with the log link, binomial family, and robust standard errors to account for clustering to obtain our parameter of interest, a prevalence ratio and 95% confidence interval (CI).

## Results

We analyzed data on all AGYW (n = 3,130); median age was 15 (interquartile range [IQR]: 14, 16), 20.2% of participants reporting being sexually active, and 28.7% reporting any FI (Table 1). Among sexually active participants, 9.3% reported ever engaging in transactional sex.

**Table 1:** Demographic characteristics of the study sample at baseline, CyberRwanda (n = 6,079)

		Total Sample (n = 6,079)	Females (n = 3,130)	Sexually Active Females (n = 633)
		N (%) or Median (SD)		
**Sex**				
Female		3130 (51.5%)	--	--
Male		2949 (48.5%)	--	--
**Age**				
12		123 (2.0%)	77 (2.5%)	12 (1.9%)
13		534 (8.8%)	320 (10.2%)	45 (7.1%)
14		1187 (19.5%)	700 (22.4%)	110 (17.4%)
15		1532 (25.2%)	840 (26.8%)	150 (23.7%)
16		1261 (20.7%)	634 (20.3%)	133 (21.0%)
17		877 (14.4%)	378 (12.1%)	115 (18.2%)
18		433 (7.1%)	155 (5.0%)	57 (9.0%)
19		132 (2.2%)	26 (0.8%)	11 (1.7%)
**Mother’s Education**				
Some primary or less		2396 (39.4%)	1251 (40.0%)	266 (42.1%)
Completed primary or more		2621 (43.2%)	1337 (42.7%)	265 (41.9%)
Unknown		1057 (17.4%)	540 (17.3%)	101 (16.0%)
**Categorical Food Security Score**				
Little to no hunger		4493 (74.0%)	2228 (71.3%)	436 (69.0%)
Moderate hunger		1464 (24.1%)	821 (26.3%)	179 (28.3%)
Severe hunger		113 (1.9%)	76 (2.4%)	18 (2.8%)
**Binary Food Security Score**				
Food Insecure	1577 (26.0%)		897 (28.7%)	197 (31.1%)
**Sexually active**				
No	4334 (71.3%)		2483 (79.3%)	0
Refuse to Answer	22 (0.4%)		14 (0.4%)	0
Yes	1723 (28.3%)		633 (20.2%)	633 (100%)
**Ever transactional sex**				
No		1590 (93.1%)	567 (90.4%)	557 (90.4%)
Yes		117 (6.9%)	60 (9.6%)	59 (9.6%)^1^

In bivariate analyses, any FI was associated with over two times the prevalence of transactional sex (unadjusted prevalence ratio [uPR]: 2.16, 95% CI: 1.37–3.41) compared to those with little to no hunger. In the second model using the three-part variable, moderate but not severe hunger were associated with a higher prevalence of transactional sex (uPR 2.11; 95% CI: 1.30–3.41; and uPR 2.72, 95% CI: 0.91–8.19, respectively) compared to the reference group (Table 2).

**Table 2:** Prevalence ratios assessing the relationship between food insecurity and ever engaging in transactional sex among female secondary students, Rwanda, 2021

	Bivariate (n = 3,106)		Adjusted (n = 3,104)		
	Prevalence ratio (95% Confidence Interval)	p-value	Prevalence ratio (95% Confidence Interval)	p-value	
**Dichotomous food security**					
Food secure	Reference		Reference		
Food insecure	2.16 (1.37, 3.41)	0.001	1.60 (1.02, 2.49)	0.039	
**Categorical food security**					
Little to no hunger	Reference		Reference		
Moderate hunger	2.11 (1.30, 3.41)	0.002	1.58 (0.99, 2.51)	0.054	
Severe hunger	2.72 (0.91, 8.19)	0.074	1.79 (0.59, 5.43)	0.30	

In the adjusted models, any experience of FI was associated with a 60% higher prevalence of transactional sex (adjusted [a]PR: 1.60, 95% CI: 1.02, 2.49). Using the three-part variable in a separate model, moderate (aPR 1.58; 95% CI: 0.99, 2.51) but not severe hunger (aPR: 1.79; 95% CI: 0.59, 5.43) was associated with transactional sex.

## Discussion

In this study, over one-quarter of in-school Rwandan AGYW reported some level of FI. While the prevalence of FI was moderately higher than the national prevalence (19%), [4] this discordance may be reflective of intra-household food reporting dynamics whereby adults tend to underestimate FI and girls generally experience higher rates of FI than other household members. [3, 17] Notably, nearly one in ten of sexually active AGYW reported ever engagement in transactional sex, and we found a positive association between FI and transactional sex such that any FI was associated with a 60% higher prevalence of transactional sex.

To our knowledge this is the first study to quantify the relationship between FI and transactional sex among Rwandan AGYW and among the first to report a statistically significant association between FI and transactional sex among in-school AGYW. Our findings that nearly 10% of in-school, sexually active girls have ever engaged in transactional sex is of great public health concern. Although there is a vast literature on the consequences of transactional sex, such as unintended pregnancy and HIV, there are fewer studies of the upstream drivers. [18, 19] Among AGYW, there are numerous *qualitative* studies documenting food scarcity as a driver for engagement in transactional sex among AGYW, [20, 21] and a small but growing body of quantitative evidence documenting drivers of transactional sex for this population. FI has been found to be an important motivator for engaging in transactional sex among AGYW aged 20–24 in South Africa [22] and AGYW aged 15–24 in Malawi. [9] Another study in South Africa found that among nulliparous AGYW (aged 11–25), being food secure was protective against transactional sex. [23] Our study is unique in that it extends this literature with its focus on in-school AGYW in the understudied Rwandan context, as well as its focus on food security. Successful interventions addressing transactional sex include cash transfers, which address its upstream drivers, such as FI; [24, 25] other behavioral interventions have not demonstrated impact. [26] Overall, these results underscore a need to mitigate poverty and FI and invest in their upstream drivers among in-school Rwandan AGYW as a means to reduce HIV transmission.

Our study purposefully chose to include girls who never engaged in sexual activity in our models and code them as never engaging in transactional sex. Given the hypothesis that food security status may predict transactional sex, if we had restricted to those who have never had sex, who by definition have also never had transactional sex, this sub-sample may suffer from selection bias, resulting in biased results. Another potential study concern is underreporting due to anticipated stigma and social desirability bias, particularly in regards to transactional sex. However, misclassification of the outcome is not a threat to the study’s validity if not dependent on food security status (i.e., non-differential misclassification); differential misclassification can bias the estimate. To understand the potential impact of underreporting of transactional sex by food insecure participants, we conducted a hypothetical exercise examining underreporting of transactional sex by 10%, 15%, and 20% among food insecure participants. In these hypothetical scenarios, the PR ranged between 4.3 and 7.0, indicating that even in the presence of differential misclassification, the estimates presented in this study may be conservative.

This study is not without limitations. There is the potential for underreporting of both FI and engagement in transactional sex; however, we demonstrated that the theoretical impact of this is minimal. Given these are cross-sectional data, we cannot disentangle temporal relationships between FI and transactional sex. The parent impact study will collect this data prospectively, which is better suited to a causal interpretation of the relationship between food security and transactional sex upon conclusion of the trial. Nevertheless, the study has multiple strengths, primarily the substantial size and representativeness of the study sample.

## Data Availability

Given that the study is ongoing, data is not available. Upon study conclusion data will be stored on the Open Science Framework site for the CyberRwanda study (https://osf.io/erd9z/).

## Declarations

### Clinical trials registry site and number

https://clinicaltrials.gov/ct2/show/NCT04198272; Registration # NCT04198272.
